# Awareness, discussion and non-prescribed use of HIV pre-exposure prophylaxis among persons living with HIV/AIDS in Italy: a Nationwide, cross-sectional study among patients on antiretrovirals and their treating HIV physicians

**DOI:** 10.1186/s12879-017-2819-5

**Published:** 2017-11-28

**Authors:** Antonio Palummieri, Gabriella De Carli, Éric Rosenthal, Patrice Cacoub, Cristina Mussini, Vincenzo Puro, the PrEPventHIV Italy Study Group, Nicoletta Ladisa, Franco Maggiolo, Marco Rizzi, Leonardo Calza, Vincenzo Colangeli, Nicolò Girometti, Alice Ferraresi, Emanuele Focà, Pier Francesco Giorgetti, Maria Chiara Pezzoli, Benedetto Maurizio Celesia, Marilia Rita Pinzone, Tiziana Bruno, Alberto Viada, Davide Vitullo, Viola Guardigni, Laura Sighinolfi, Silvia Ambu, Dario Bartolozzi, Irene Campolmi, Massimo Meli, Marco Pozzi, Gaetana Sterrantino, Filippo Matarazzo, Francesco Purificato, Enza Anzalone, Luciano Sarracino, Miriam Lichtner, Raffaella Marocco, Vito Sante Mercurio, Paula Castelli, Lorena Paoli Martorelli, Angela Linzalone, Franklyn Esoka Eseme, Enzo Raise, Simona Bossolasco, Antonella Castagna, Massimo Cernuschi, Paola Cinque, Luca Fumagalli, Giovanni Gaiera, Nicola Gianotti, Monica Guffanti, Miriam Maillard, Silvia Nozza, Vincenzo Spagnuolo, Caterina Uberti-Foppa, Vanni Borghi, Cristina Mussini, Luchino Chessa, Laura Matta, Maria Cristina Pasetto, Giuseppe D’Esposito, Alfredo Franco, Crescenzo Maria Izzo, Elio Manzillo, Alessandro Marocco, Raffaele Micillo, Teresa Pizzella, Salvatore Martini, Maria Vittoria Moretti, Elisabetta Schiaroli, Corrado Catalani, Marina Giorgi, Beatrice Menichini, Michele Trezzi, Simona Migliore, Giuseppe Ballardini, Enrico Barchi, Elisa Garlassi, Giacomo Magnani, Francesca Prati, Lucia Testa, Maria Alessandra Ursitti, Giuliana Zoboli, Elisabetta Teti, Anna Rita Buonomini, Carlotta Cerva, Giovanni D’Anna, Barbara Ilari, Vincenzo Malagnino, Loredana Sarmati, Adriana Ammassari, Rita Bellagamba, Evangelo Boumis, Stefania Cicalini, Giuseppina Liuzzi, Laura Loiacono, Paolo Migliorisi, Emanuele Nicastri, Carmela Pinnetti, Alessandro Sampaolesi, Chiara Tommasi, Mauro Zaccarelli, Rosalia Angileri, Maria Coscia, Gabriella d’Ettorre, Alessandra Fantauzzi, Giancarlo Iaiani, Ivano Mezzaroma, Francesca Paoletti, Maria Zaccaria, Andrea De Luca, Barbara Rossetti, Manola Pomi, Nicoletta Carnicelli, Angela Gonnelli, Daniele Marri, Lucia Toscano, Orlando Armignacco, Anna Rita Capino, Antonio Luciano Caterini, Barbara Di Filippo, Anna Maria Ialungo, Elena Rastrelli, Rita Sabatini, Gabriella De Carli, Antonio Palummieri, Vincenzo Puro

**Affiliations:** 1 National Institute for Infectious Diseases “Lazzaro Spallanzani” IRCCS, Department of Epidemiology, Pre-clinical Research and Advanced Diagnostics, via Portuense, 292, 00149 Roma, Italy; 2 Centre Hospitalier Universitaire de Nice, Service de Médecine Interne, F-06200 Nice, France; 3 Sorbonne Universités, UPMC Univ Paris 06, UMR 7211, and Inflammation-Immunopathology-Biotherapy Department (DHU i2B), F-75005 Paris, France; 4 INSERM, UMR_S 959, F-75013 Paris, France; 5 CNRS, FRE3632, F-75005 Paris, France; 6 AP-HP, Groupe Hospitalier Pitié-Salpêtrière, Department of Internal Medicine and Clinical Immunology, F-75013 Paris, France; 7 Università degli Studi di Modena e Reggio Emilia, Infectious Diseases Clinics, Modena, Italy

**Keywords:** Pre-Exposure Prophylaxis (PrEP), HIV prevention, Anti-HIV agents, Persons Living with HIV/AIDS (PLWHA), HIV physicians

## Abstract

**Background:**

Before Pre-Exposure Prophylaxis (PrEP) was officially recommended and made available, a few surveys among gay and bisexual men, and persons living with HIV/AIDS (PLWHA), identified an informal use of antiretrovirals (ARVs) for PrEP among HIV-negative individuals. Before PrEP availability in Italy, we aimed to assess whether PLWHA in Italy shared their ARVs with HIV-negative individuals, whether they knew people who were on PrEP, and describe the level of awareness and discussion on this preventive measure among them and people in their close circle.

**Methods:**

Two anonymous questionnaires investigating personal characteristics and PrEP awareness, knowledge, and experience were proposed to HIV specialists and their patients on ARVs in a one-week, cross-sectional survey (December 2013–January 2014). Among PLWHA, a Multivariable Logistic Regression analysis was conducted to identify factors associated with PrEP *discussion with peers* (close circle and/or HIV associations), and *experience* (use in close circle and/or personal ARV sharing).

**Results:**

Eighty-seven specialists in 31 representative Infectious Diseases departments administered the questionnaire to 1405 PLWHA. Among specialists, 98% reported awareness, 65% knew the dosage schedule, and 14% had previously suggested or prescribed PrEP. Among PLWHA, 45.6% were somehow aware, discussed or had direct or indirect experience of PrEP: 38% “had heard” of PrEP, 24% were aware of studies in HIV-negative individuals demonstrating a risk reduction through the use of ARVs, 22% had discussed PrEP, 12% with peers; 9% reported PrEP use in close circle and 1% personal ARV sharing. Factors predictive of either PrEP discussion with peers or experience differed between men and women, but across all genders were mainly related to having access to information, with HIV association membership being the strongest predictor.

**Conclusions:**

At a time and place where there were neither official information nor proposals or interventions to guide public policies on PrEP in Italy, a significant number of PLWHA were aware of it, and approximately 10% reported PrEP use in their close circle, although they rarely shared their ARVs with uninfected people for this purpose. Official policies and PrEP availability, along with implementation programs, could avoid risks from uncontrolled PrEP procurement and self-administration practices.

**Electronic supplementary material:**

The online version of this article (10.1186/s12879-017-2819-5) contains supplementary material, which is available to authorized users.

## Background

The efficacy and safety of pre-exposure prophylaxis (PrEP) with oral tenofovir disoproxil fumarate (TDF) with or without emtricitabine (FTC) to prevent human immunodeficiency virus (HIV) infection were clearly demonstrated in randomized, blinded and placebo-controlled trials, among men who have sex with men (MSM) and transgender women, sexually active heterosexual adults, and intravenous drug users (IVDU) [1–4]. Two European studies launched in 2012 gave further evidence that daily or on demand PrEP confers high protection against HIV in MSM when adherence is consistent [5, 6].

On the basis of the resulting high-quality evidence, in 2012 the U.S. Food and Drug Administration (FDA) and in 2016 the European Medicines Agency (EMA) approved tenofovir-emtricitabine (TDF–FTC, Truvada®) for PrEP in adults at high risk for contracting HIV infection, and guidelines were issued recommending that oral PrEP (containing TDF) should be offered as an additional prevention choice for people at substantial risk of HIV infection as part of combination prevention approaches [7–9].

However, outside the U.S. widespread uptake has not been immediate. In Europe PrEP is not routinely recommended and prescribed, and as of January 2017, Truvada® for PrEP has been available only in France, following a Temporary Recommendation for Use by the French regulatory agency (ANSM), and in Switzerland.

Data from the U.S., Australia and France, however, suggested an informal use of antiretrovirals (ARVs) for PrEP among HIV-negative individuals before PrEP was officially recommended and made available, as identified through surveys of gay and bisexual men, and persons living with HIV/AIDS (PLWHA) [10–12].

The National Institute for Infectious Diseases “L. Spallanzani” (INMI) in Rome is the coordinating centre of the Italian Registry of Antiretroviral Post-Exposure Prophylaxis (IRAPEP), and is supporting studies and initiatives to introduce PrEP in national prevention plans. Therefore, before PrEP was recommended and available in Italy, a nationwide survey was launched by INMI, to assess whether PLWHA in Italy shared ARV for PrEP with uninfected people, and describe awareness and discussion on PrEP in this population.

## Methods

### Study design

At the INMI, we adopted the design, and translated and adapted the questionnaires (Additional file 1 a-d), used for the PREVIC cross-sectional study conducted in France [12], kindly provided by the PREVIC coordinators. The survey involved PLWHA and their treating HIV physicians in hospital and university/research departments and Institutes of infectious diseases in Italy. Before the beginning of the study, the translated questionnaires were pre-tested at INMI on patients participating in the mutual help group at the Psychology Unit and on seven physicians of the AIDS Referral Unit, all not participating in the study afterwards.

Physicians in HIV centers providing ARV treatment were contacted by e-mail during the first week of October 2013. They could participate in the study enrolling through an online form.

Each specialist was invited to propose an anonymous standardized questionnaire (Additional file 1 a-b) to all PLWHA on ARVs for at least 3 months, consecutively visited between 9 and 14 December 2013 or, if the specialist was unavailable in that week, between 13 and 18 January 2014. The questionnaire for PLWHA included socio-demographic characteristics (gender, age group, place of residence, socio-economic category), whether the responder was a member or supporter of an association for the fight against AIDS (HIV association), and risk behaviors and clinical, virological and immunological status of the participants. A second section included eight closed questions on PrEP, regarding: awareness; discussion with their close circle (which could include stable or occasional partners, immediate family members, close friends, or community members), physician or members of HIV associations; use in their close circle or sharing personal ARVs for PrEP. The questionnaire took approximately 10 min to be completed. The patients received no incentives to participate in the survey.

A second standardized questionnaire (Additional file 1 c–d) was addressed to the participating HIV physicians to obtain individual data (gender, age, and type of institution), whether the responder was a member or supporter of an association for the fight against AIDS, outpatients seen during the study week, and knowledge and previous prescription of PrEP. Both questionnaires were anonymously completed, and each paper form was then placed in a sealed envelope and sent to the principal investigator. The datasets were enclosed (Additional file 2 a-b).

The study was approved by the INMI Ethics Committee (n.56/2013), and by the ECs of the San Raffaele Hospital (Milan), Fondazione Policlinico Tor Vergata (Rome), Area Vasta Sud Est (Siena), Aziende Sanitarie Umbria (Perugia); the remaining centres adopted the INMI’s EC resolution.

### Statistical analysis

Participating PLWHA were stratified according to gender. We conducted univariate analyses to identify factors associated with PrEP discussion and experience (χ^2^, Fisher exact test), defined as follows: *PrEP discussion* grouped discussion either with persons in the PLWHA close circle or members of HIV associations, or both, considered as peers; *PrEP experience* grouped use of PrEP either in the PLWHA close circle or personal ARV sharing, or both.

A Multivariable Logistic Regression (MLR) analysis was obtained through stepwise backward elimination, including the variables that showed significance in the univariate model (*p* < .10). Gender and age group were possibly forced into each regression model, and three models (overall, female –F, male-M) were built. Transgender PLWHA, representing around 1% of the overall sample, were only included in the overall model. Statistical analysis was performed with IBM® SPSS® Statistics version 21.0 (Chicago, IL).

## Results

### HIV physicians and patients

Eighty-seven HIV physicians from 31 representative departments of Infectious Diseases in 13 Regions participated in the study (centers participation rate, 22.2%); 56% were from a research institute/university. Women represented 53%; most physicians were aged > 50 (55%), 70% were unit heads or staff. Eight percent were member of a HIV association. Physicians reported awareness of PrEP (98%), with 65% reporting knowledge of the dosage schedule. Twelve (14%) had previously suggested (*n* = 10) or prescribed (*n* = 2) PrEP, in serodiscordant couples for conception (*n* = 9), subjects with multiple partners (n = 1), or in case of nonconsistent condom use (n = 2), with tenofovir alone (n = 1) or Truvada® (*n* = 11).

These HIV physicians saw a median number of 23 outpatients during the study week (range 2–92). Overall, they proposed the study to 1506 PLWHA: 63 refused and 38 were eliminated because of inconsistencies in the questionnaire. Of the 1405 PLWHA eventually enrolled, they all filled the whole questionnaire: 71.8% were males (HIV transmission route: homosexual intercourse 43%, heterosexual intercourse 25%, IVDU 17%, not reported-NR 15%), 27% females (heterosexual intercourse 59%, homo-bisexual intercourse 6%, IVDU 15%, NR 20%), 1.2% transgender. Altogether, 17% were member of HIV associations. Most (80%) reported undetectable viral load; 24% were coinfected with hepatitis C virus. Eight percent reported a sexually-transmitted infection over the last 12 months. Over the last 3 months, 68% (*n* = 961) reported sexual intercourse, 16% (*n* = 226) with multiple partners; 19% did not use condoms with their stable uninfected partner or with casual partners.

### Knowledge, discussion and use of PrEP

“Having heard” of PrEP was reported by 539 PLWHA (38.4%), but of these, 337 were actually aware of studies in HIV negative individuals demonstrating a risk reduction through the use of ARVs (24%). These were more frequently member of HIV associations (27% vs 14%; OR 2.28, CI 95% 1.68–3.10, *p* < .001), and used condoms with their stable partner (54% vs 43%; OR 1.56, 1.21–2.01, *p* < .001).

Among all participants, 305 (21.7%) discussed PrEP with someone, 166 of whom (12% of the whole population) reported discussion with individuals of their close circle or members of HIV associations. Regarding experience, 126 participants (9%) reported PrEP use in their close circle, and 14 (1%) personal ARV sharing. Only 58 individuals (4.1%; 42 males, 3 transgender, 13 females) overlapped between the two groups, having reported either discussion with peers and use in close circle or ARV sharing. Most persons reporting either discussion or PrEP experience were males (75%), MSM (45%), aged > 40 (65%), lived in a metropolitan area (50%) and had a single partner over the last 3 months (50%).

In the MLR model, the following factors were found to be associated with a greater likelihood of discussing PrEP with peers: HIV association membership (All), sexual activity (multiple partners, All), age group (30–40 years, M-model), route of HIV transmission (homo/bisexual intercourse, IVDU, M-model), occupation (employee/intellectual worker, F-model), CD4 count (< 200/mm^3^, F-model), and viral load (detectable, F-model) (Table 1).

**Table 1:** Multivariable logistic regression analyses predicting PrEP discussion with peers

	ALL GENDERS^a^ *N* = 166/1405				FEMALE^b^ *n* = 43/379				MALE^c^ *n* = 119/1009			
	N (%)	AOR	(95% CI)	*p*-value	n (%)	AOR	(95% CI)	*p*-value	n (%)	AOR	(95% CI)	*p*-value
Age (years)												
< 30	12 (16.7)	1.5	0.7–2.9						8 (17.8)	1.3	0.5–3.0	0.568
30–40	45 (16.7)	1.6	1.0–2.3	0.034					32 (18.1)	1.7	1.1–2.8	0.026
> 40	109 (10.2)	1							79 (10.0)	1		
Socio-economic category												
Without occupation	30 (11.3)	1			7 (6.2)	1			21 (14.8)	1		
Farmers/Intermediary workers	47 (9.4)	0.9	0.5–1.4	0.659	12 (10.7)	1.7	0.6–4.7	0.316	33 (8.5)	0.6	0.3–1.1	0.087
Employee/Intellectual workers	72 (16.9)	1.7	1.0–2.7	0.036	19 (17.8)	3.8	1.4–10.2	0.007	53 (16.7)	1.2	0.6–2.1	0.608
Retired	17 (8.1)	0.8	0.4–1.6	0.549	5 (10.6)	1.7	0.5–6	0.395	12 (7.3)	0.6	0.3–1.3	0.189
Member of HIV associations												
Yes	51 (21.3)	2.4	1.6–3.5	<0.001	11 (19.0)	2.4	1.1–5.4	0.028	37 (21.1)	2.4	1.6–3.8	<0.001
Undetectable HIV RNA												
No					14 (20.3)	2.5	1.1–5.3	0.023				
Route of HIV transmission												
Heterosexual intercourse	41 (8.7)	1							19 (7.7)	1		
Homo/Bisexual intercourse	74 (15.8)	1.7	1.1–2.6	0.015					66 (15.4)	1.8	1.0–3.1	0.041
Intravenous Drug Use	32 (14.0)	2.0	1.2–3.4	0.008					22 (12.9)	2.0	1.0–4.0	0.038
Other/unknown	19 (7.9)	1.0	0.5–1.7	0.967					12 (7.4)	1.0	0.5–2.2	0.915
CD4 count/mm^3^												
< 200	21 (17.9)	2.1	1.2–3.7	0.009	7 (19.4)	2.9	1.0–8.5	0.050				
200–499	45 (12.0)	1.2	0.8–1.8	0.325	12 (12.5)	1.5	0.7–3.6	0.308				
500+	75 (11.2)	1			16 (8.2)	1						
NR/ND	25 (10.4)	1.2	0.7–2.0	0.501	8 (15.4)	2.1	0.8–5.8	0.144				
Partners’ number (last 3 months)												
No partner	39 (8.8)	1			14 (9.2)	1			25 (8.6)	1		
Single partner	86 (11.7)	1.2	0.8–1.8	0.490	24 (11.2)	1.3	0.6–2.7	0.545	61 (11.8)	1.3	0.8–2.1	0.347
Multiple partners	41 (18.1)	1.7	1.0–2.9	0.042	5 (45.5)	8.9	2.1–37.7	0.003	33 (16.4)	1.6	0.9–3.0	0.096

*AOR* adjusted odds ratio, *CI* confidence interval, *ART* antiretroviral therapy, *STI* sexually transmitted infection

^a^Including Transgender persons (*n* = 4/17). Significant variables at univariate analysis (*p* < 0.100): Gender (forced variable); Age; Socio-economic category; Member of HIV associations; Route of HIV transmission; CD4 count/mm^3^; STI (last 12 months); Partners’ number (last 3 months)

^b^Significant variables at univariate analysis (*p* < 0.100): Age (forced variable); Socio-economic category; Member of HIV associations; Undetectable HIV RNA; CD4 count/mm^3^; Partners’ number (last 3 months)

^c^Significant variables at univariate analysis (*p* < 0.100): Age; Place of residence; Socio-economic category; Member of HIV associations; Route of HIV transmission; Partners’ number (last 3 months)

Factors associated with a greater likelihood of reporting PrEP experience were: gender (transgender, male), HIV association membership (All), younger age (< 30, 30–40 years, M-model), route of HIV transmission (homo/bisexual intercourse, IVDU, M-model), ARV changes over the last 12 months (M-model), sexual activity (multiple partners, F-model), enrolling institution (research institute, F-model), and viral load (detectable, F-model) (Table 2).

**Table 2:** Multivariable logistic regression analyses predicting PrEP experience

	ALL GENDERS^a^ *N* = 140/1405				FEMALE^b^ *n* = 24/379				MALE^c^ *n* = 111/1009			
	N (%)	AOR	(95% CI)	*p*-value	N (%)	AOR	(95% CI)	*p*-value	N (%)	AOR	(95% CI)	*p*-value
Gender												
Female	24 (6.3)	1			–	–			–	–		
Male	111 (11.0)	1.5	0.9–2.6	0.088	–	–			–	–		
Transgender	5 (29.4)	3.4	1.0–11.4	0.049	–	–			–	–		
Age (years)												
< 30	12 (16.7)	2.1	1.1–4.2	0.031					9 (20.0)	2.3	1.0–5.0	0.048
30–40	33 (12.3)	1.6	1.0–2.5	0.034					25 (14.1)	1.8	1.1–3.0	0.026
> 40	95 (8.9)	1							77 (9.8)	1		
Enrolling centre												
General hospital	32 (7.3)	1			3 (2.5)	1						
Research Institute	57 (13.5)	1.9	1.2–3.1	0.006	12 (11.2)	5.5	1.4–21.6	0.014				
University Institute	51 (9.4)	1.3	0.8–2.1	0.244	9 (5.9)	2.3	0.6–9.1	0.250				
Member of HIV associations												
Yes	42 (17.5)	2.2	1.5–3.3	<0.001	6 (10.3)	2.6	0.9–7.6	0.074	33 (18.9)	2.2	1.4–3.4	0.001
Route of HIV transmission												
Heterosexual intercourse	27 (5.7)	1							16 (6.5)	1		
Homo/Bisexual intercourse	57 (12.2)	1.7	1.0–2.8	0.061					49 (11.4)	1.6	0.9–3.0	0.109
Intravenous Drug Use	34 (14.8)	2.6	1.5–4.5	0.001					29 (17.0)	3.1	1.6–5.9	0.001
Other/unknown	22 (9.2)	1.5	0.8–2.8	0.172					17 (10.4)	1.7	0.8–3.5	0.151
Change ART (last 12 months)												
Yes									32 (14.5)	1.5	0.9–2.3	0.102
Undetectable HIV RNA												
No	39 (13.7)	1.6	1.1–2.5	0.018	10 (14.5)	4.5	1.8–11.3	0.002				
Partners’ number (last 3 months)												
No partner	36 (8.1)				7 (4.6)	1						
Single partner	70 (9.5)				13 (6.0)	1.5	0.6–4.0	0.421				
Multiple partners	34 (15.0)				4 (36.4)	17.5	3.6–84.9	<0.001				

*AOR* adjusted odds ratio, *CI Confidence Interval*, *ART* antiretroviral therapy, *STI* sexually transmitted infection

^a^Including Transgender persons (*n* = 5/17). Significant variables at univariate analysis (*p* < 0.100): Gender; Age; Enrolling centre; Member of HIV associations; Route of HIV transmission; Undetectable HIV RNA; Partners’ number (last 3 months)

^b^Significant variables at univariate analysis (*p* < 0.100): Age (forced variable); Enrolling centre; Member of HIV associations; Route of HIV transmission; Undetectable HIV RNA; CD4 count/mm^3^; STI (last 12 mos); Partners’ number (last 3 months)

^c^Significant variables at univariate analysis (*p* < 0.100): Age; Enrolling centre; Member of HIV associations; Route of HIV transmission; Change ART (last 12 months)

## Discussion

In spite of the lack of public information or regulation on PrEP in Italy at the time of this study, 38% of the participants were aware of PrEP, 24% knew the results of the studies demonstrating a risk reduction through the use of ARVs, 22% had discussed PrEP with their peers or doctors, 9% had people in their close circle who used PrEP, and 1% had shared their personal ARVs for PrEP with someone.

Though the study was conducted in Italy 2 years after the PREVIC Study, after FDA approved Truvada® for PrEP in adults at high risk for contracting HIV infection, and after the launch of two well-publicized PrEP studies in Europe, these proportions are very similar to those observed in France [12].

However, only some similarities were observed in the two PLWHA populations (French and Italian) regarding factors associated with a greater likelihood of discussing PrEP with peers or having PrEP experience: among males, HIV association membership and non-heterosexual route of HIV transmission; and among females, having multiple partners. Actually, in Italy, HIV association membership was the strongest predictor of discussing and having a personal or a close experience of PrEP across the whole patient population (males, females and transgender persons), thereby confirming the crucial role that associations play in the spread of information [13]. Indeed, in our study, factors predictive of either discussion or experience regarding PrEP seem mostly related to having access to information on this issue, especially among females. Other factors seem to be related with the possibility of transmitting the infection, i.e., having multiple partners among all genders, route of HIV transmission among males, and having a detectable viral load among females, though in a previous study, the fear of infecting the partner among women living with HIV in Italy was unrelated with virological control in plasma [14].

Also very similar were the proportions of French and Italian HIV physicians aware of PrEP, knowing the dosage schedule, and having actually prescribed PrEP. Data on PrEP knowledge, though not further assessed, are consistent with previous results among Italian HIV specialists [15]. Among patients, discussion with their HIV physician was reported by 15%, though we excluded this item from the MLR analysis as in Italy we had observed conflicting results regarding attitudes towards PrEP prescription among HIV physicians [15, 16].

These results cannot be considered fully representative of the whole population on ARVs in Italy, even if the centres which contributed are among those which treat the highest proportion of PLWHA. However, our results are in line with those of the “Flash PrEP” survey conducted almost contemporarily to characterize informal PrEP use in France [17]. Moreover, a significant increase in awareness and attitude is likely to have occurred after the widely publicized appearance of the results from IPERGAY [5] and PROUD [6], the international guidelines, and the EMA approval, so that any further delay in incorporating PrEP in national prevention protocols might result in dangerous practices. HIV seroconversion while using non-prescribed ARVs for PrEP has been reported, and uncontrolled use might undermine the protective benefits of PrEP [18].

As a biomedical HIV prevention technology, PrEP opens up opportunities for expanded autonomy in managing one’s own sexual health, and might re-balance structural asymmetries through shifting the control of sexual risk from the HIV-positive to the HIV-negative partner, a fact especially important for women [19]. PrEP also prefigures a different kind of user of sexual health services, one more mobile and active than the traditional idea of patient allows [20]. Therefore, it should not be unexpected that 45.6% of PLWHA were somehow aware, discussed or had direct or indirect experience of PrEP at a time and place where there were neither information nor proposals or interventions to guide public policies in this regard. This underlines that the temporal rhythms of connection between affected communities and public health systems should be synchronized to avoid uncontrolled procurement practices and self-administration of ARVs amongst HIV-exposed persons.

## Conclusions

These results suggest that although PrEP is not officially provided in Italy, a significant number of PLWHA are aware of it, and approximately 10% report PrEP use in their close circle. While PLWHA seem to rarely share their ARVs with uninfected people for this purpose, associations representing the LGBT Community recently launched an alert regarding “online shopping” and the consequent lack of appropriate clinical and serological monitoring [21]. In the absence of national implementation programs, these findings alert towards the risks deriving from uncontrolled PrEP procurement and self-administration practices, and call for official policies on PrEP offer and use at a country level.

## Additional files

Additional file 1: a: Questionario per i pazienti: original questionnaire (Italian version) on Pre-Exposure Prophylaxis awareness, discussion and practice for Persons Living With HIV/AIDS; b: English version. c: Questionario per i medici: original questionnaire (Italian version) on Pre-Exposure Prophylaxis awareness, discussion and practice for HIV Specialists caring for Persons Living With HIV/AIDS; 1d: English version. (ZIP 958 kb)

Additional file 2: a: PrEPventHIV_patients_1405. Original database (Patients). b: PrEPventHIV_physicians_87. Original database (HIV specialists). (ZIP 177 kb)

## Abbreviations

ANSM: Agence Nationale de Sécurité du Médicament et des Produits de Santé
ARVs: Antiretrovirals
EC: Ethics Committee
EMA: European Medicines Agency
F: Female
FDA: Food and Drug Administration
FTC: Emtricitabine
HIV: Human immunodeficiency virus
INMI: Istituto Nazionale Malattie Infettive (National Institute for Infectious Diseases) “L. Spallanzani”
IVDU: Intravenous drug users
M: Male
MLR: Multivariable Logistic Regression
MSM: Men who have sex with men
NR: Not reported
PLWHA: Persons living with HIV/AIDS
PrEP: Pre-Exposure Prophylaxis
TDF: Tenofovir disoproxil fumarate
