# Supplementary material for: Awareness, discussion and non-prescribed use of HIV pre-exposure prophylaxis among persons living with HIV/AIDS in Italy: a Nationwide, cross-sectional study among patients on antiretrovirals and their treating HIV physicians
Source: BMC Infect Dis. 2017 Nov 28;17:734. doi: 10.1186/s12879-017-2819-5 (PMC5704632; doi:10.1186/s12879-017-2819-5)
Supplement: Supplementary file 1 — a: Questionario per i pazienti: original questionnaire (Italian version) on Pre-Exposure Prophylaxis awareness, discussion and practice for Persons Living With HIV/AIDS; b: English version. c: Questionario per i medici: original questionnaire (Italian version) on Pre-Exposure Prophylaxis awareness, discussion and practice for HIV Specialists caring for Persons Living With HIV/AIDS; 1d: English version. (ZIP 958 kb) [file 12879_2017_2819_MOESM1_ESM.zip › additional file 1/Supplementary file 1aR3.pdf]

Codice medico
___________

# STUDIO NAZIONALE PREVIC 2013

## *INCHIESTA SULLE PRATICHE DI PROFILASSI PRE-ESPOSIZIONE*

***Risponda solo se ha iniziato la terapia antiretrovirale da più di 3 mesi***

**Se rifiuta di rispondere al questionario, per quale ragione:**
- ☐ Mancanza di tempo ☐ Non mi riguarda ☐ Non sono interessato(a)
- ☐ Altra ragione:______________________

**Sesso**: ☐ Donna ☐ Uomo ☐ Transgender

**Età**: ☐ < 30 anni ☐ 30-40 anni ☐ 41-50 anni ☐ 51-60 anni ☐ > 60 anni

**Luogo di residenza**:
- ☐ Area metropolitana e/o periferie (Milano, Torino, Genova, Bologna, Venezia, Firenze, Roma, Napoli, Bari, Reggio Calabria, Trieste, Messina, Catania, Palermo, Cagliari)
- ☐ Area Urbana (città non compresa nelle aree metropolitane)
- ☐ Area Rurale (< di 5.000 abitanti)
- ☐ Altro______________________

**Categoria socio-professionale:**
- ☐ Agricoltore
- ☐ Dirigente
- ☐ Operaio
- ☐ Pensionato
- ☐ Artigiano, Commerciante, Imprenditore
- ☐ Professione medica o paramedica
- ☐ Impiegato
- ☐ Senza attività

**È membro, o simpatizzante di un'associazione per la lotta contro l'AIDS?** ☐ Sì ☐ No

**Come si è contagiato con HIV?**
- ☐ Rapporti omo/bisessuali ☐ Rapporti eterosessuali ☐ Uso di droghe per via endovenosa
- ☐ Trasmissione materno-fetale ☐ Trasfusioni ☐ Indeterminato o altro

**Il suo medico infettivologo ha cambiato il suo piano terapeutico antiretrovirale negli ultimi 12 mesi?**
☐ Sì ☐ No

**La sua infezione HIV è sotto controllo (carica virale/viremia non rilevabile)?** ☐ Sì ☐ No

**Ultimo valore dei CD4/mm$^3$:**
- ☐ <100 ☐ 100-200 ☐ 201-350
- ☐ 351-500 ☐ > 500 ☐ Non lo so

**La sua infezione HIV ha necessitato di un'ospedalizzazione almeno una volta in questi ultimi 12 mesi?**
☐ Sì ☐ No

**È coinfetto con**:
- *epatite C*: ☐ Si ☐ No
- *epatite B*: ☐ Si ☐ No

**Negli ultimi 12 mesi, ha contratto un'infezione sessualmente trasmissibile (sifilide, condilomi, herpes genitale…)?**
☐ Sì ☐ No

**Ha un partner stabile?** ☐ Sì ☐ No

**Se sì, il suo partner è sieronegativo?** ☐ Sì ☐ No

**Usa regolarmente il preservativo con il suo partner stabile?** ☐ Sì ☐ No

**Usa regolarmente il preservativo con i partner occasionali?** ☐ Sì ☐ No

**Negli ultimi 3 mesi, ha avuto rapporti sessuali?** ☐ Sì ☐ No

**Se sì, con quanti partner?** ☐ 1 partner ☐ più di un partner

**Negli ultimi 3 mesi, ha avuto uno o più rapporti sessuali penetrativi e senza protezione?** ☐ Sì ☐ No

*Le domande successive riguardano la Profilassi Pre-Esposizione che consiste nella somministrazione di farmaci antiretrovirali a persone sieronegative (HIV negative) prima dei rapporti sessuali per proteggerle dall'infezione da HIV*

| | | | |
|---|---|---|---|
| 1. | **Ha già sentito parlare della Profilassi Pre-Esposizione?** | ☐ Sì | ☐ No |
| 2. | **Conosce studi che abbiano dimostrato come l'utilizzo di antiretrovirali in persone non HIV positive riduca il rischio di infezione da HIV?** | ☐ Sì | ☐ No |
| 3. | **Ha parlato negli ultimi tre mesi con persone della sua cerchia di conoscenti/amici della profilassi pre-esposizione anti-HIV?** | ☐ Sì | ☐ No |
| 4. | **Ha parlato negli ultimi tre mesi con persone dell'ambiente associativo della profilassi pre-esposizione anti-HIV?** | ☐ Sì | ☐ No |
| 5. | **Ha parlato negli ultimi tre mesi con il suo medico della profilassi pre-esposizione anti-HIV?** | ☐ Sì | ☐ No |
| 6. | **Conosce nella sua cerchia di conoscenti/amici una o più persone che abbiano fatto ricorso a farmaci antiretrovirali come profilassi pre-esposizione anti-HIV?** | ☐ Sì | ☐ No |
| 7. | **Negli ultimi tre mesi qualcuno le ha chiesto di condividere uno o più farmaci antiretrovirali come profilassi pre-esposizione anti-HIV?** | ☐ Sì | ☐ No |
| 8. | **Negli ultimi tre mesi ha condiviso uno o più dei suoi farmaci antiretrovirali con qualcuno che desiderava utilizzarli come profilassi pre-esposizione anti-HIV?** | ☐ Sì | ☐ No |

## Grazie per la sua collaborazione
