# Supplementary material for: Awareness, discussion and non-prescribed use of HIV pre-exposure prophylaxis among persons living with HIV/AIDS in Italy: a Nationwide, cross-sectional study among patients on antiretrovirals and their treating HIV physicians
Source: BMC Infect Dis. 2017 Nov 28;17:734. doi: 10.1186/s12879-017-2819-5 (PMC5704632; doi:10.1186/s12879-017-2819-5)
Supplement: Supplementary file 1 — a: Questionario per i pazienti: original questionnaire (Italian version) on Pre-Exposure Prophylaxis awareness, discussion and practice for Persons Living With HIV/AIDS; b: English version. c: Questionario per i medici: original questionnaire (Italian version) on Pre-Exposure Prophylaxis awareness, discussion and practice for HIV Specialists caring for Persons Living With HIV/AIDS; 1d: English version. (ZIP 958 kb) [file 12879_2017_2819_MOESM1_ESM.zip › additional file 1/Supplementary file 1bR3.pdf]

PREVIC 2013 NATIONAL STUDY

Survey on Pre-Exposure Prophylaxis Practices

Please answer only if you have been on antiretroviral therapy for at least 3 months

If you refuse to answer, for which reason:
- ☐ Lack of time ☐ It does not concern me ☐ I am not interested
- ☐ Other:______________________

Gender: ☐ Female ☐ Male ☐ Transgender

Age group: ☐ < 30 years ☐ 30-40 years ☐ 41-50 years ☐ 51-60 years ☐ > 60 years

Place of residence:
- ☐ Metropolitan Areas and related suburban areas (Milano, Torino, Genova, Bologna, Venezia, Firenze, Roma, Napoli, Bari, Reggio Calabria, Trieste, Messina, Catania, Palermo, Cagliari)
- ☐ Urban Area (cities not included in metropolitan areas)
- ☐ Rural Area (< di 5.000 inhabitants)
- ☐ Other______________________

Socio-economic category:
- ☐ Farmer
- ☐ Executive
- ☐ Workman
- ☐ Retired
- ☐ Craftsman, tradesman, businessman
- ☐ Medical or paramedical profession
- ☐ Employee
- ☐ Unemployed

Are you a member of an HIV/AIDS patients' association? ☐ Yes ☐ No

How do you think you have acquired HIV infection?
- ☐ Homo-bisexual intercourse ☐ Heterosexual intercourse ☐ Intravenous drug use
- ☐ Vertical transmission ☐ Transfusions ☐ Unknown/other

Did your HIV specialist change your antiretroviral therapy over the last 12 months?
☐ Yes ☐ No

Did you reach HIV suppression (undetectable viral load)? ☐ Yes ☐ No

Last CD4 level: ☐ <100 ☐ 100-200 ☐ 201-350
☐ 351-500 ☐ > 500 ☐ Don't know

Were you hospitalized because of HIV-related conditions over the last 12 months?
☐ Yes ☐ No

Are you coinfected with: *C hepatitis* : ☐ Yes ☐ No

B hepatitis : ☐ Yes ☐ No

Did you acquire a sexually-transmitted infection (syphilis, herpes, gonorrhea..) over the last 12 months?
☐ Yes ☐ No

Do you have a stable partner? ☐ Yes ☐ No

If you do, is your partner HIV negative? ☐ Yes ☐ No

Do you regularly use condoms with your stable partner? ☐ Yes ☐ No

Do you regularly use condoms with casual partners? ☐ Yes ☐ No

In the last three months, did you have sex? ☐ Yes ☐ No

If you did, with how many partners?
☐ 1 partner
☐ more than 1 partner

In the last three months, did you have one or more penetrating and unprotected sexual intercourse?
☐ Yes ☐ No

The following questions concern Pre-Exposure Prophylaxis, which consists in the administration of antiretroviral drugs to HIV-negative individuals prior to sexual intercourse to protect them from HIV infection

**1. Have you ever heard of Pre-Exposure Prophylaxis (PrEP)?**	☐ Yes	☐ No
**2. Are you aware of studies in HIV negative individuals demonstrating a reduction of HIV risk through the use of antiretrovirals?**	☐ Yes	☐ No
**3. Did you discuss PrEP in your close circle (partners, close friends, family or community members) in the last three months?**	☐ Yes	☐ No
**4. Did you discuss PrEP with members of HIV associations in the last three months?**	☐ Yes	☐ No
**5. Did you discuss PrEP with your HIV physician in the last three months?**	☐ Yes	☐ No
**6. Do you know people in your close circle who used antiretrovirals as PrEP?**	☐ Yes	☐ No
**7. Over the last three months, have you been asked to share your antiretrovirals for PrEP?**	☐ Yes	☐ No
**8. Over the last three months, did you share your antiretrovirals as PrEP?**	☐ Yes	☐ No

<u>Thank you for your cooperation</u>
