# Supplementary material for: Awareness, discussion and non-prescribed use of HIV pre-exposure prophylaxis among persons living with HIV/AIDS in Italy: a Nationwide, cross-sectional study among patients on antiretrovirals and their treating HIV physicians
Source: BMC Infect Dis. 2017 Nov 28;17:734. doi: 10.1186/s12879-017-2819-5 (PMC5704632; doi:10.1186/s12879-017-2819-5)
Supplement: Supplementary file 1 — a: Questionario per i pazienti: original questionnaire (Italian version) on Pre-Exposure Prophylaxis awareness, discussion and practice for Persons Living With HIV/AIDS; b: English version. c: Questionario per i medici: original questionnaire (Italian version) on Pre-Exposure Prophylaxis awareness, discussion and practice for HIV Specialists caring for Persons Living With HIV/AIDS; 1d: English version. (ZIP 958 kb) [file 12879_2017_2819_MOESM1_ESM.zip › additional file 1/Supplementary file 1cR3.pdf]

Allegato C

STUDIO NAZIONALE PREVIC 2013

INCHIESTA *SULLE PRATICHE DI PROFILASSI PRE-ESPOSIZIONE*

QUESTIONARIO MEDICI

1. Sesso: ☐ Donna ☐ Uomo

2. Età: ☐ < 30 anni ☐ 30-40 anni ☐ 41-50 anni ☐ 51-60 anni ☐ > 60 anni

3. Quali sono gli orientamenti principali del suo reparto: ☐ Medicina interna ☐ Malattie Infettive ☐ Immunologia ☐ Dermatologia/venereologia ☐ Malattie Tropicali ☐ Altro (precisare):

4. Tipo di struttura: ☐ IRCCS ☐ Ospedale Universitario ☐ Ospedale pubblico/Azienda ospedaliera

5. Ruolo: ☐ Dirigente medico II livello/Primario ☐ Dirigente medico I ☐ Specializzando ☐ Contrattista ☐ Frequentatore ☐ Ricercatore

6. È membro di un'associazione di pazienti che si adopera nel settore HIV/AIDS? ☐ Sì ☐ No

Attività medica svolta in questa ultima settimana

- Numero totale di pazienti visti in visita ambulatoriale: |__|__|__|
- Numero di pazienti HIV visti in visita ambulatoriale: |__|__|__|
- Numero totale di pazienti visti in day hospital: |__|__|__|
- Numero di pazienti HIV visti in day hospital: |__|__|__|

Profilassi Pre-Esposizione

1. Conosce la Profilassi Pre-Esposizione, che consiste nel somministrare un farmaco antiretrovirale a persone sieronegative per proteggerle dall'infezione da HIV? ☐ Sì ☐ No

2. Se sì, conosce le modalità di prescrizione? ☐ Sì ☐ No

3. Ha già consigliato o prescritto negli ultimi sei mesi una Profilassi Pre-Esposizione? ☐ Sì ☐ No

a. Se sì, con quali molecole?

☐ Atripla ☐ Viread ☐ Kivexa ☐ Truvada
☐ Epivir ☐ Ziagen ☐ Sustiva ☐ Kaletra
☐ Reyataz ☐ Prezista ☐ Isentress ☐ Altro____________________

b. Se sì, con quale schema terapeutico?

☐ Dose unica ☐ Trattamento continuo ☐ Trattamento discontinuo (specificare):____________

c. Se sì, in quali circostanze? (più risposte possibili)

☐ Coppia sierodiscordante ☐ Desiderio di procreazione
☐ Partner multipli ☐ Non utilizzo di alcun metodo di prevenzione
☐ Altro (specificare):________________________________

Grazie per la sua collaborazione

CONFIDENZIALE
