# Supplementary material for: Awareness, discussion and non-prescribed use of HIV pre-exposure prophylaxis among persons living with HIV/AIDS in Italy: a Nationwide, cross-sectional study among patients on antiretrovirals and their treating HIV physicians
Source: BMC Infect Dis. 2017 Nov 28;17:734. doi: 10.1186/s12879-017-2819-5 (PMC5704632; doi:10.1186/s12879-017-2819-5)
Supplement: Supplementary file 1 — a: Questionario per i pazienti: original questionnaire (Italian version) on Pre-Exposure Prophylaxis awareness, discussion and practice for Persons Living With HIV/AIDS; b: English version. c: Questionario per i medici: original questionnaire (Italian version) on Pre-Exposure Prophylaxis awareness, discussion and practice for HIV Specialists caring for Persons Living With HIV/AIDS; 1d: English version. (ZIP 958 kb) [file 12879_2017_2819_MOESM1_ESM.zip › additional file 1/Supplementary file 1dR3.pdf]

PREVIC 2013 NATIONAL STUDY

Pre-Exposure Prophylaxis Practices

Physician questionnaire

1. Gender: ☐ Female ☐ Male

2. Age group: ☐ < 30 years ☐ 30-40 years ☐ 41-50 years ☐ 51-60 years ☐ > 60 years

3. Working Unit/Department:
☐ Internal Medicine ☐ Infectious Diseases
☐ Immunology ☐ Dermatology/Venereal Diseases
☐ Tropical Diseases ☐ Other (specify):____________

4. Type of institution: ☐ Research Institute ☐ University Hospital ☐ Non-teaching Hospital

5. Role: ☐ Head ☐ Resident ☐ Graduate student ☐ Contract (temporary) doctor ☐ Attending physician ☐ Researcher

6. Are you a member of an HIV/AIDS patients' association? ☐ Yes ☐ No

Clinical activity carried out in the study week

- Total outpatients visited: |__|__|__|
- Total HIV outpatients visited: |__|__|__|

- Total patients visited in day hospital: |__|__|__|
- Total HIV patients visited in day hospital |__|__|__|

Pre-Exposure Prophylaxis

1. Do you have knowledge of Pre-Exposure prophylaxis, which consists of administering an antiretroviral drug to seronegative people to protect them from HIV infection? ☐ Yes ☐ No

☐ **If so, do you know how to prescribe it?** ☐ Yes ☐ No

2. Did you already *(choose one of the following)* ☐ **suggest** ☐ **prescribe pre-exposure prophylaxis over the last 6 months?**

☐ Sì ☐ No

a. If you did, which drugs did you suggest/prescribe?
☐ Atripla ☐ Viread ☐ Kivexa ☐ Truvada
☐ Epivir ☐ Ziagen ☐ Sustiva ☐ Kaletra
☐ Reyataz ☐ Prezista ☐ Isentress ☐ Other__________________

b. If you did, which schedule did you suggest/prescribe?
☐ Single dose (before or after a sexual intercourse)
☐ Daily dose
☐ On demand (please describe):_________________

c. If you did, in which circumstances? (multiple choice)
☐ Serodiscordant couple ☐ Family planning
☐ Multiple partners ☐ Not using condoms or other preventive methods
☐ Other (please describe):_________________

Thank you for your cooperation
